# Enhanced Ultrafast Broadband Reverse Saturable Absorption in Twistacenes with Enlarged π-Conjugated Central Bridge

**DOI:** 10.3390/molecules27249059

**Published:** 2022-12-19

**Authors:** Xindi Liu, Wenfa Zhou, Mengyi Wang, Xingzhi Wu, Jidong Jia, Jinchong Xiao, Junyi Yang, Yinglin Song

**Affiliations:** 1 School of Physical Science and Technology, Soochow University, Suzhou 215006, China; 2 Department of Physics, Harbin Institute of Technology, Harbin 150001, China; 3 Jiangsu Key Laboratory of Micro and Nano Heat Fluid Flow Technology and Energy Application, School of Physical Science and Technology, Suzhou University of Science and Technology, Suzhou 215009, China; 4 Key Laboratory of Chemical Biology of Hebei Province, College of Chemistry and Environment Science, Hebei University, Baoding 071002, China

**Keywords:** twistacenes, nonlinear optics, Z-scan, excited state absorption, optical power limiting

## Abstract

Optical nonlinearities of two all-carbon twistacenes, **DPyA** and **DPyN**, with the different π-conjugated central bridges were investigated. The nonlinear absorption properties of these compounds were measured using the femtosecond Z-scan with wavelengths between 650 and 900 nm. It has been found that the nonlinear absorption originated from two-photon absorption (TPA) and TPA-induced excited state absorption (ESA), wherein **DPyA** demonstrates higher performance than **DPyN**. The TPA cross section of **DPyA** (4300 GM) is nearly 4.3 times larger than that of **DPyN** at 650 nm. Moreover, the different central structures modulate the intensity of ESA at 532 nm, and **DPyA** exhibits an excellent ESA at 532 nm with multi-pulse excitation. Meanwhile, the result of data fitting and quantum chemistry calculation shows that the enhancement of nonlinear absorption in **DPyA** is due to the extended π- conjugated bridge and improved delocalization of π-electrons. These all-carbon twistacenes could yield potential applications in optical power limiting (OPL) technology.

## 1. Introduction

With the rapid development of laser technology, the ultrafast laser is widely used in various fields, such as laser processing, laser shaping, medical imaging, and all-optical switcher [1,2,3,4]. Many kinds of optical power limiting (OPL) materials that protect human eyes and precise sensors from laser damage are synthesized and optimized, such as fullerene derivatives [5,6], nanoparticles [7,8,9], and metal-organic compounds [10], etc. [11,12]. Some disadvantages of OPL materials, such as their suitability only for long-pulse-duration lasers and a narrow limiting region, degrade the OPL performance. TPA, as the ultrafast broadband absorption process, is regarded as an ideal molecular transition approach to realize the OPL response during ultrashort pulses [13,14,15]. Moreover, the cooperative amplification of TPA and TPA-induced ESA over a wide response range may be feasible for improving OPL performance [15].

Twistacenes, an important part of polycyclic aromatic hydrocarbons (PAHs), inhibit π-π stacking collectively induced by their extended conjugation, which leads to the formation of electron-deficient backbone coupled with its strong steric hindrances at the peripheral edges. Benefitted from excellent thermal, chemical, and light stability, in addition to easy modification and significant photoelectric performance, twistacenes are widely used as active layers in photoelectric materials [16,17,18,19]. When one pyrene unit is connected to acene, the formed twistacene has advantageous broadband ESA [20]. It is well-known that, compared with monomers, branched structures not only effectively decrease the intramolecular and intermolecular stacking interaction in both the solution and solid state, but also incorporate multiple TPA enhancement factors into the molecular system to further enhance the TPA and nonlinear optical (NLO) properties [19,21]. Previous work of our groups has demonstrated that the π-conjugated central bridge in the symmetric twistacenes plays a key role in modulating TPA and TPA-induced ESA. Moreover, the extension of π-conjugation length enlarges ESA in a twistacene [20,22]. In addition to the highly delocalized electrons and lower system energy (e.g., the energy of the molecule with a five-membered ring is lower as compared to that of the benzene ring) related to the size of the central bridge, these structures are easy-to-synthesize, stable, and demonstrate high third-order polarizability [23,24]. These favorable features of twistacenes inspire us to find more excellent central π- bridges to optimize NLO properties.

**Scheme 1:**
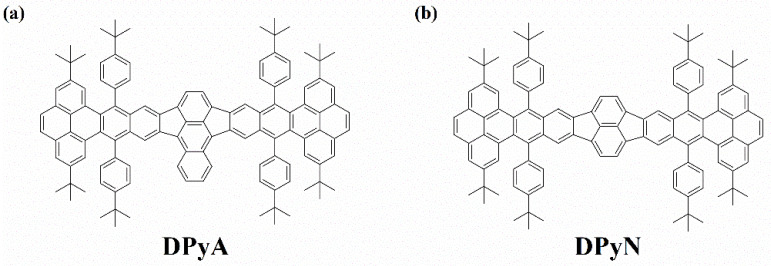
Structural diagrams of **DPyA** (**a**) and **DPyN** (**b**).

In this work, the structure–property relationship of two twistacenes are studied: 2,7-di-tert-butyl-9,14-bis(4-(tert-butyl)phenyl)dibenzo[de,qr]tetracene-fused naphthalene (**DPyN**) and anthracene (**DPyA**), respectively (Scheme 1), which were previously synthesized by Xiao et al. [25]. The central bridges of **DPyN** and **DPyA** feature naphthalene and anthracene, respectively. Based on quantum chemical calculation, the ground state optimized structure and electron−hole distribution of these two compounds were extracted. In addition, Z-scan measurements at various wavelengths for different pulse widths were utilized to evaluate the NLO response. Transient absorption (TA) spectroscopy was performed to analyze the evolution process of ESA of excitons at high energy levels.

## 2. Results and Discussion

### 2.1. UV-Vis Absorption and Emission Spectra

**Figure 1:**
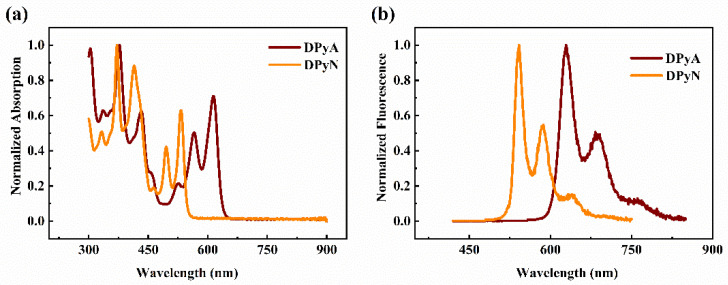
UV-vis absorption (**a**) and emission (**b**) spectra of **DPyA** and **DPyN**.

The UV-vis absorption and emission spectra of **DPyA** and **DPyN** were obtained (Figure 1) at room temperature in toluene solution with the concentration of 1 × 10^−5^ M. Several absorption peaks at 376 nm, 432 nm, 565 nm, and 614 nm can be found in the UV-vis absorption spectrum of **DPyA**. When compared to the absorption spectrum of **DPyN** (495 nm and 533 nm), the absorption peak of **DPyA** shows a clear red shift of nearly 82 nm. The red shift of the absorption in **DPyA** is due to the extension of the π-conjugation length in the bridge. Both twistacenes demonstrate similar emission properties (Figure 1b). The fluorescence spectrum of **DPyA** covers the wide spectral range from 600 nm to 750 nm and exhibits two peaks at 629 nm and 688 nm. The changes in the fluorescence spectrum are consistent also with those in the absorption spectrum. The maximum fluorescence peak of **DPyA** shows a redshift of 88 nm against that of **DPyN** (541/586 nm). The fluorescence lifetimes were further extracted as 4.6 ns for **DPyA** and 3.4 ns for **DPyN,** respectively (Figure S1), by means of time-correlated single-photon counting (TCSPC). Two studied twistacenes have excellent thermal and photostability [25], which indicates that these two molecules are potential candidates for technical applications.

### 2.2. Transient Absorption Measurements

**Figure 2:**
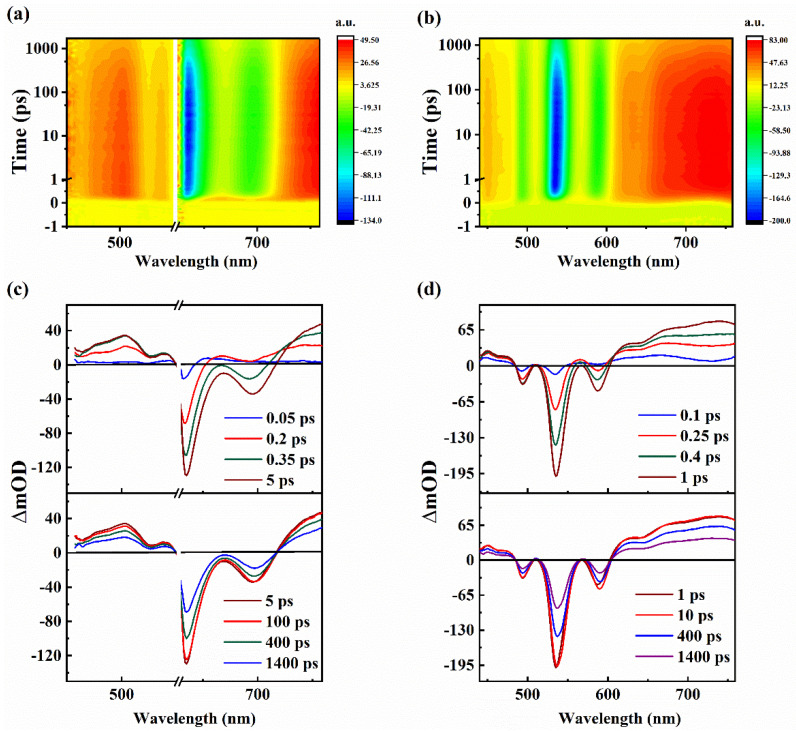
TA spectra of **DPyA** (**a**) and **DPyN** (**b**). The curves of TA spectra of **DPyA** (**c**) and **DPyN** (**d**) are shown for a specific delay time.

To reveal the evolution process of ESA of excitons at high energy levels with the femtosecond laser excitation, the TA spectra of both samples were obtained at a pumping wavelength of 400 nm and a probe window (440–900 nm) ranging from visible (Figure 2a,b) to near-infrared (Figure S2) region. Several TA spectra of the compounds DPyA and **DPyN** for specific delay time are shown in Figure 2c,d. The change of absorption intensity, extracted from the TA at a particular wavelength, is expressed as the change in optical density (Δ*OD*) that could be calculated as follows: $\Delta OD=-\mathrm{lg}\left(T/T_{0}\right)$. Herein, *T* and *T*_0_ are the transmittances of the sample after pumping and after no pumping, respectively.

For **DPyA** (Figure 2a,c), two positive signals (450–550 nm and 640–760 nm, respectively) and one negative signal (centered at 635 nm) start to form near zero delay time. As the delay time increases, an ultrafast positive signal (650–710 nm) changes to a negative signal at 0.2 ps. Then, all signals are becoming more intense. The signals reach their maximum values at about 5 ps. Within the next 100 ps, the intensity of signals displays a tiny variation. Finally, the intensities of all signals maintain similar attenuation tendencies. The positive signal in the TA spectra represents ESA, while the negative signal denotes saturable absorption (SA). Herein, the negative signal with peaks at 628 and 686 nm may be ascribed to stimulated emission (SE), which corresponds with emission spectra. Meanwhile, the other negative signal (centered at 566/614 nm) could be caused by ground state bleaching. The change of a signal may denote the cooperation between ESA and SE. Compared with **DPyA**, there are some distinct features in the TA spectrum of **DPyN**. The negative and positive signals of **DPyN** have a blue shift, which could be caused by the shift of UV-vis absorption peaks and emission peaks. It is easy to find that peak shapes in the TA spectra of two studied twistacenes exhibit no evident variation with the probe time delay, so we can assume that ESA may be a singlet state absorption.

**Figure 3:**
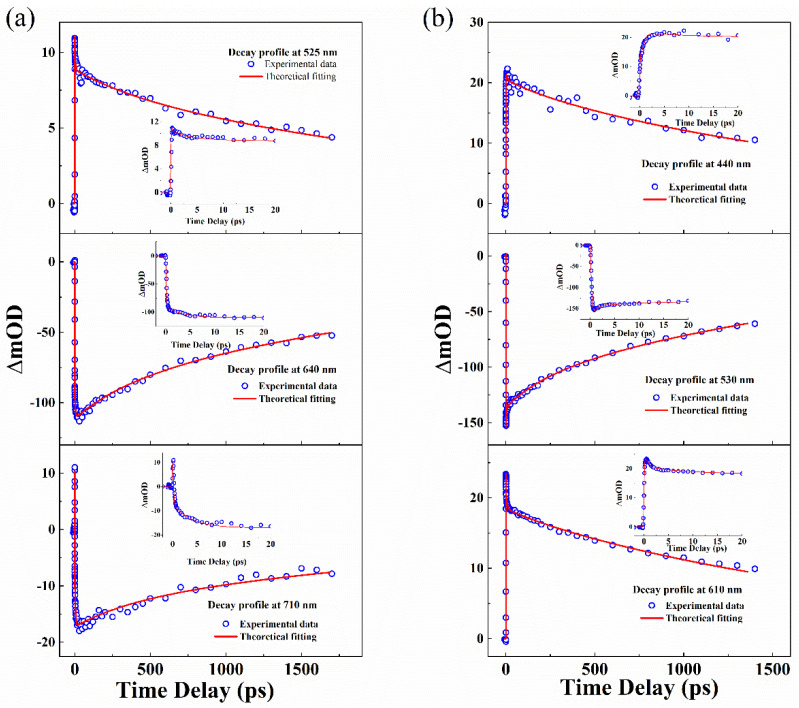
The dynamic curves of selected wavelengths for **DPyA** (**a**) and **DPyN** (**b**), excited at 400 nm. The illustration shows the same early stage (within the first 20 ps).

**Figure 4:**
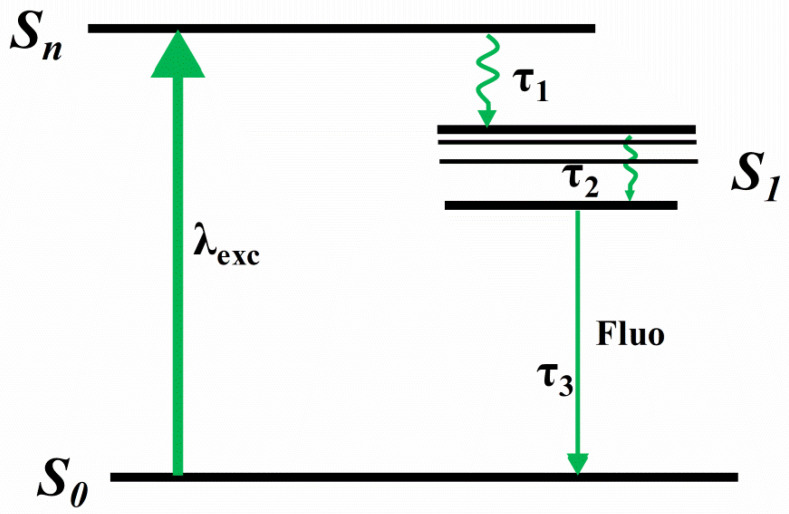
The relaxation processes of excitons absorbing high photon energy.

To analyze the evolution process of the TA spectra, which shows that the molecules are in their ground state and absorbs energy of pumped photons and exhibits different relaxation processes, dynamic TA curves of **DPyA** and **DPyN** were fitted with the global fitting analysis (shown in Figure 3) by means of a sequential model [26]. In total, three dynamic processes have been recognized and obtained their lifetimes, which are summarized in Table S1. As shown in Figure 4, we have defined a simplified relaxation model to demonstrate the relaxation processes of excitons.

In general, under photoexcitation, the molecule in the ground state absorbs the energy of the pump light and then populates to a higher excited state *S_n_*. There are three processes of excitons returning to ground state. (1) The first process can be regarded as the establishment of *S_n_* and the lifetime of *S_n_* is *τ*_1_. (2) After several picoseconds, there is no obvious change in the whole spectrum that indicates the establishment of *S*_1_, and the lifetime of vibrational cooling relaxation in the *S*_1_ is *τ*_2_. (3) The final long relaxation process (*τ*_3_) can be regarded as conformational relaxation from the *S*_1_ to *S*_0_ accompanying radiative transition. On the other hand, the lifetime (*τ*_3_) matches the results of fluorescence lifetime, indirectly indicating ESA work mostly in the singlet excited state.

### 2.3. Femtosecond Nonlinear Optical Absorption

**Figure 5:**
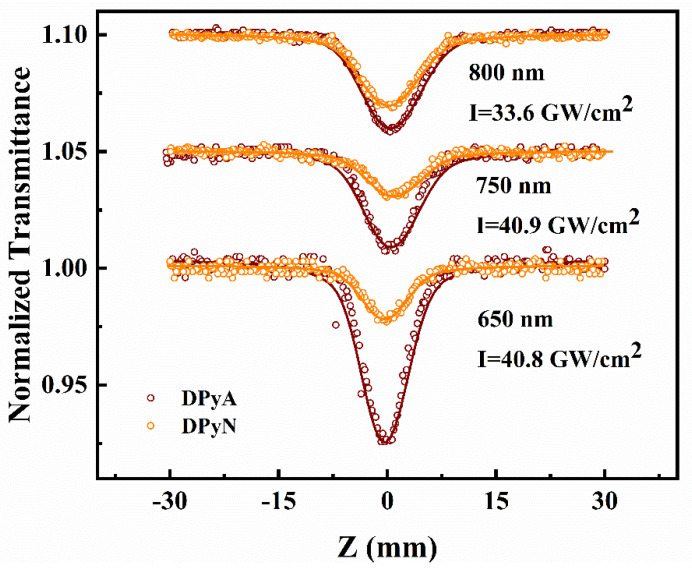
Open-aperture Z-scan experiment of twistacenes **DPyA** and **DPyN** under 190 fs at 650 nm, 750 nm, and 800 nm. The circles are measured data. The solid lines represent theoretical fitting.

To obtain a good knowledge of the observed optical nonlinearities and to estimate the NLO performance, femtosecond open-aperture Z-scan experiments with different wavelengths (650/700/750/800/850/900 nm) were conducted. Both compounds exhibit high transmittance at all wavelengths (≥98%, **DPyA** at 650 nm is 88%). Here, three wavelengths (650/750/800 nm) were selected as examples shown in Figure 5. A single-valley at z = 0 (focus point) represents reverse saturable absorption (RSA). By fitting with Sheik Bahae’s theory [27], we extracted the effective NLA coefficient β_eff_ which is shown in Table S2. It can be found that the strength of RSA in **DPyA** is better than **DPyN** at all measured wavelengths.

**Figure 6:**
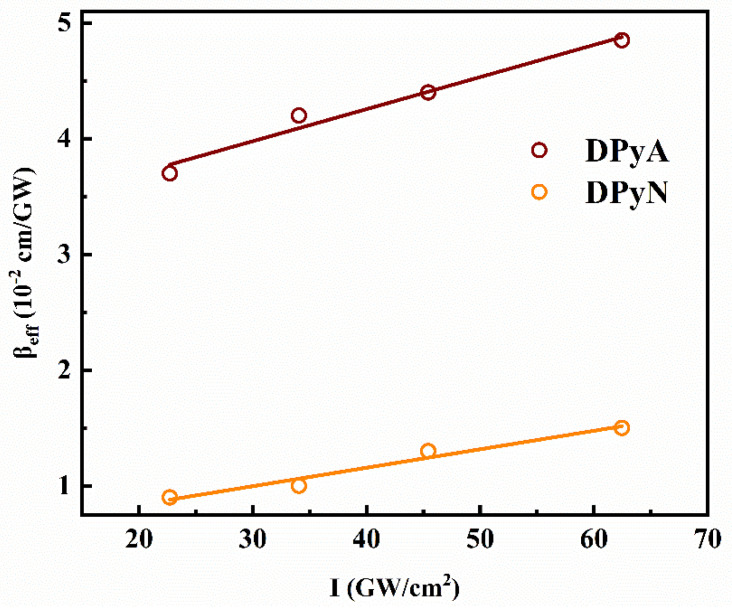
The effective nonlinear absorption coefficient is a function of input peak intensity for twistacenes **DPyA** and **DPyN** at 650 nm.

Generally, TPA and ESA are the main reasons for the RSA. The high transmittance in the experimental wavelengths and the transition energies (*S*_0_–*S*_1_) of the molecule is 2.30 eV for **DPyA** and 2.72 eV for **DPyN** (shown in Figure S3). The photon energies between 650 nm and 900 nm are 1.91–1.38 eV. Therefore, the NLA mainly originates from TPA. To further analyze the NLO mechanism of both compounds, open-aperture Z-scan experiments under different peak intensities at various wavelengths (650/700/750/800/850/900 nm) are conducted. All the measurement data are shown in Figures S4 and S5. The relationship between β_eff_ and input peak intensity I at 650 nm is shown in Figure 6 as an example. β_eff_ of both compounds increase linearly with I, which suggests that TPA is not the only cause of RSA. Combined with the TA spectrum, the higher-order NLA can be attributed to TPA-induced ESA. TPA and TPA-induced ESA may simultaneously exhibit in the organic molecules under fs photoexcitation [19,22,28,29].

In general terms, TPA-induced ESA could be considered fifth-order nonlinear absorption. Under photoexcitation, the molecule in the ground state simultaneously absorbs two photons to leap to the excited state. Then, the excitons in the excited state absorb one photon to populate in a higher excited state. To obtain TPA cross sections $\sigma_{TPA}$ and ESA cross sections $\sigma_{S_{1}}$, the model obtained in TA was used for numerical fitting. The simplified rate equation can be given by:

(1) $$\left.\begin{array}{l} \frac{dN_{S_{0}}}{dt}=-\frac{\sigma_{S_{0}}IN_{S_{0}}}{\hbar \omega }-\frac{\sigma_{TPA}N_{S_{0}}I^{2}}{2\hbar^{2}\omega^{2}}+\frac{N_{S_{1}}}{\tau_{S_{1}}}\\ \frac{dN_{S_{1}}}{dt}=\frac{\sigma_{S_{0}}IN_{S_{0}}}{\hbar \omega }-\frac{\sigma_{S_{1}}N_{S_{1}}I}{\hbar \omega }-\frac{N_{S_{1}}}{\tau_{S_{1}}}+\frac{N_{S_{n}}}{\tau_{S_{n}}}\\ \frac{dN_{S_{n}}}{dt}=\frac{\sigma_{TPA}N_{S_{0}}I^{2}}{2\hbar^{2}\omega^{2}}+\frac{\sigma_{s_{1}}IN_{s_{1}}}{\hbar \omega }-\frac{N_{s_{n}}}{\tau_{S_{n}}} \end{array}\right\}$$

The light inside the sample can be calculated using

(2) $$\frac{dI}{dz}=-\left(\sigma_{S_{0}}N_{S_{0}}+\sigma_{S_{1}}N_{S_{1}}+\frac{\sigma_{TPA}N_{S_{0}}I}{\hbar \omega }\right)I$$

where $N_{S_{0}},N_{S_{1}},N_{S_{n}}$ are the population density populated at *S*_0_, *S*_1,_ and *S_n_*. $\sigma_{TPA}$ and $\sigma_{S_{1}}$ are the two photon and excited state absorption cross sections, respectively. $\tau_{S_{1}}$ and $\tau_{S_{n}}$ represent the lifetime of excited state *S*_1_ and *S_n_*, which can be roughly extracted from TA spectra. ℏ and ω stand for the constant of reduced Planck and angular frequency, respectively. The fitting results are shown in Figures S4 and S5 and the parameters are listed in Table 1.

Both molecules are extremely large. The $\sigma_{TPA}$ of **DPyA** is larger than **DPyN** at all wavelengths. Especially at 650 nm, the $\sigma_{TPA}$ of **DPyA** and **DPyN** are 4300 GM and 1000 GM, respectively. The $\sigma_{TPA}$ of **DPyA** is 4.3 times larger than **DPyN**. The minimum $\sigma_{TPA}$ ratio of the two compounds is about 1.8 at 900 nm. The ratio index of $\sigma_{TPA}$ to molecular molar mass ($\sigma_{TPA}$/M) [22] of **DPyA** is 2.8 at 650 nm, which is four times larger than that of **DPyN** ($\sigma_{TPA}$/M = 0.68). It is very beneficial to practical applications. This observation suggests that a large enhancement of NLA can be achieved with only a slight increase in molecular weight at the π-central bridge.

**Figure 7:**
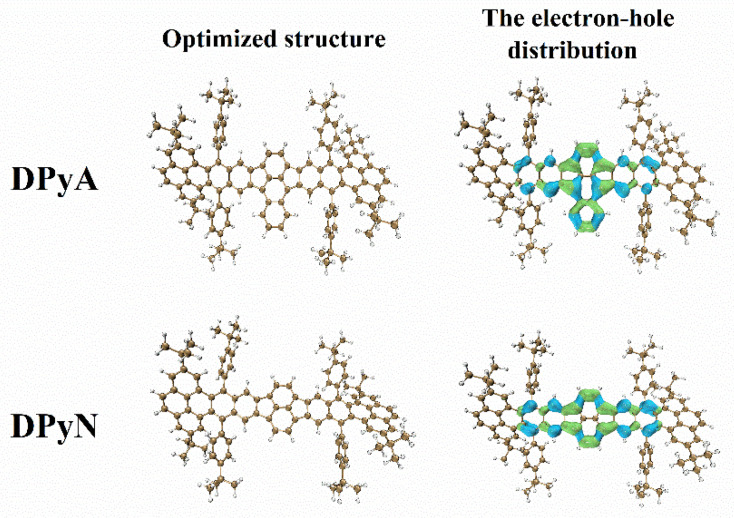
The ground state optimized structure and electron-hole distribution of **DPyA** and **DPyN** extracted from DFT and TD-DFT.

In order to find the structure–property relationship between π-electron excitation properties and NLO response, the frontier molecular orbital distribution (shown in Figure S7) and the electron-hole distribution (Figure 7) were calculated. As shown in Figure 7, the green represents the increase of electrons, and the blue represents the decrease of the electrons. The distributions of electron–hole are mainly localized in the two five-member rings, the adjacent fused benzene and the naphthalene group (for **DPyN**) and anthracene group (for **DPyA**), indicating that both molecules have typical π-π * transitions during the TPA process. This distribution shows that most of the central skeleton of the molecule works in the NLO response. Compared with the structure of **DPyN**, **DPyA** has an extra benzene in the center, which provides more delocalized electrons. In general, molecular planarity not only increased the effective π-conjugation structure of the molecule but also enhanced the charge delocalization extent. As can be seen from the optimization structure shown in Figure 7, both **DPyA** and **DPyN** have a good planar central skeleton. To our knowledge, molecules with good planarity and many delocalized electrons exhibit superior TPA performance. This may be the main reason that the $\sigma_{TPA}$ of **DPyA** is larger than that of **DPyN**.

In addition, compared to other organic compounds, the TPA cross section ($\sigma_{TPA}$) of **DPyA** at 650 nm is an excellent improvement compared to the previously reported nonlinear compounds (Table 2).

### 2.4. Nonlinear Optical Absorption Properties at Various Pulse-Widths

**Figure 8:**
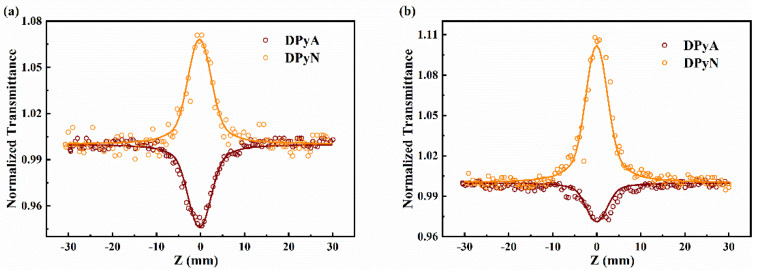
Open-aperture Z-scan experiment of twistacenes **DPyA** and **DPyN** under 22 ps (**a**) and 4 ns (**b**) at 532 nm. The circles are measured data. The solid lines represent the theoretical fitting.

To study the NLO response of **DPyA** and **DPyN** at different pulse-widths, we performed open-aperture Z-scan experiments at 532 nm using 22 ps and 4 ns laser pulses. The **DPyA** exhibits RSA in both picosecond and nanosecond (Figure 8) while **DPyN** shows SA. The β_eff_ values (Table 3) are obtained by Sheik Bahae’s theory [27]. The open-aperture Z-scan experiment data of **DPyA** and **DPyN** with different input peak intensities are shown (Figures S6 and S7). The transmittance of **DPyA** is 67% and that of **DPyN** is 61%. Due to the 532 nm in the resonance region, the RSA of **DPyA** derives from the ESA in accordance with the results of TA at 532 nm. The more benzene in the center enhances the conjugation degree of the system and modulates the intensity of ESA at 532 nm. Therefore, these two compounds show opposite properties. These phenomena provide a new angle to better understand the structure–property relationship and also provide a possibility for further optimization of molecular structure.

## 3. Experimental Section

### 3.1. Ultrafast Time-Resolved Absorption Spectra

The procedures of the ultrafast TA measurement (Figure S8) are detailed in Ref [38]. A mode-locked Yb:KGW-based fiber laser (PHAROS, Light Conversion, FWHM:190 fs, 1030 nm) was used as the light source, and then the pump beam was output by an optical parametric amplifier (OPA, ORPHEUS). The pump beam wavelength was tuned to 400 nm. The pump beam fluence was measured to be 11 mW. In addition, the probe beam is supercontinuum white light produced by focusing the portion of the source light on a sapphire. The probe spectral region of 440~760 nm was recorded by rotatable grating inside the spectrometer.

### 3.2. Open-Aperture Z-Scan Experiment

Open-aperture Z-scan is the general method for investigating nonlinear absorption (NLA) properties (Figure S9) [27]. Femtosecond open-aperture Z-scan measurements at various wavelengths were carried out. Both compounds were dissolved in toluene at the concentration of 0.5 mg/mL and placed in the 2 mm quartz cell. The light source was consistent with that used in the TA experiment. Moreover, measurements were carried out with different pulse widths at 532 nm: 22 picosecond pulses from a Q-switched Nd:YAG laser (BKAZER-2P, GRACE LASER), and 4 nanosecond pulses from a Q-switched Nd:YAG laser (Surelite II, Continuum). To exclude the thermal effect, the repetition frequency of the input pulse was tuned to 20 Hz for fs Z-scan, and 10 Hz for ps, ns Z-scan. The solvent did not exhibit NLA at all experimental laser intensities.

### 3.3. DFT Calculation

To explain the different properties of the two studied twistacenes, quantum chemical calculation was perfomed. Density functional theory (DFT), with B3LYP functional and 6–31G (d,p) basic set [39,40], was employed to optimize the ground state geometric structure. Time-dependent density functional theory (TD-DFT) with CAM-B3LYP functional and 6–31G (d,p) basic set [41] were utilized to obtain the electron characteristics of excited states. The calculation of ground state and excited states was performed by the Gaussian 09 package [42]. The electron-hole distribution [43] was analyzed by Multimfn 3.8 and the VMD program [44].

## 4. Conclusions

In summary, we systematically investigated the NLO properties of two twistacenes with different π-central bridges, **DPyN** and **DPyA**. The open-aperture Z-scan at various wavelengths under fs laser excitation shows that both twistacenes have excellent RSA properties, which are derived from TPA and TPA-induced ESA. The RSA performance of compound **DPyA** is better than **DPyN**. Using the distribution of electron-hole, better RSA properties may be caused by more delocalized electrons in the central π-bridge. Moreover, $\sigma_{TPA}$ of **DPyA** is 4300 GM, 4.3-times larger than **DPyN** at 650 nm. The minimum $\sigma_{TPA}$ ratio of the two compounds is about 1.8 at 900 nm. Meanwhile, the more benzene in the center modulates the intensity of ESA at 532 nm and the two compounds show the opposite properties at 532 nm under multi-pulse excitation. TA spectra also provide ultrafast dynamics analysis of ESA in high energy levels, revealing the relaxation process. These two twistacenes can be potential candidates used in OPL applications.

## Figures and Tables

**Table 1:** The NLA of twistacenes **DPyA** and **DPyN** at different wavelengths were selected by femtosecond Z-scan.

	DPyA		DPyN	
Wavelength (nm)	$\sigma_{TPA}$ (GM)	$\sigma_{S_{1}}$ 10^−21^ m^2^	$\sigma_{TPA}$ (GM)	$\sigma_{S_{1}}$ 10^−21^ m^2^
650	4300	7	1000	22
700	3500	8	900	20
750	2300	7	850	17
800	1500	5	650	13
850	900	3	420	10
900	600	2	330	9

**Table 2:** Comparison of the TPA cross section between twistacene **DPyA** and **DPyN** with some reported excellent NLO Materials.

Sample	Wavelength (nm)	$\sigma_{TPA}$ (GM)	Journal	Ref
**DPyA**	650	4300		This work
BTAN-TPOC5-COF	800	1124	*Small*	[30]
3333 m-MTDATA:TPBi	750	387	*J. Lumin.*	[31]
bisbtz-VI	830	1252	*Org. Lett.*	[32]
7 PDI-(OPh)_4_	710	595	*Dyes Pigments*	[33]
AIE NPs	800	560	*ACS Nano.*	[34]
cyano-sp^2^c-COF	800	1225	*Adv. Funct. Mater.*	[35]
1a	730	696	*Angew. Chem. Int. Ed. Engl.*	[36]
TCzP-BDTO	820	693	*Adv.Funct. Mater.*	[37]

**Table 3:** The NLA coefficient of twistacenes **DPyA** and **DPyN** at different pulse-widths were extracted by picosecond and nanosecond Z-scans.

Sample	DPyA ^a^	DPyN ^a^	DPyA ^b^	DPyN ^b^
β_eff_ (cm/GW)	0.7	-	10.5	-
I_S_ (10^−3^ GW/cm^2^)	-	45	-	5

^a^ at 532 nm with the 22 ps laser excitation. ^b^ at 532 nm with the 4 ns laser excitation.

## Data Availability

The data presented in this study are available in Supplementary Materials.

## Supplementary Materials

The following supporting information can be downloaded at: https://www.mdpi.com/article/10.3390/molecules27249059/s1. Figure S1: Fluorescence decay data of **DPyA** (a) and **DPyN** (b) by TCSPC. The solid lines were fitted lifetimes of 4.6 ns for **DPyA** and 3.4 ns for **DPyN**. Figure S2: The TA spectra of **DPyA** at the visible window (a) and near the infrared window (b) excited at 400 nm. The TA spectra of **DPyN** at the visible window (c) and near the infrared window (d) excited at 400 nm. Figure S3: The frontier molecular orbital distributions of **DPyA** and **DPyN** extracted from DFT calculation. Figure S4: Open-aperture Z-scan results of **DPyA** at different excitation wavelengths (650 nm, 700 nm, 750 nm, 800 nm, 850 nm, and 900 nm). Figure S5: Open-aperture Z-scan results of **DPyN** at different excitation wavelengths (650 nm, 700 nm, 750 nm, 800 nm, 850 nm, and 900 nm). Figure S6: Open-aperture Z-scan experiment at 532 nm under 22 ps laser width for **DPyA** (a) and **DPyN** (b). Figure S7: Open aperture Z-scan experiment at 532 nm under 4 ns laser width for **DPyA** (a) and **DPyN** (b). Figure S8: Schematic illustration of transient absorption system. Figure S9: Schematic illustration of the Z-scan system. Scheme S1: Synthetic route to molecules **DPyA** and **DPyN**. Table S1: Fitting parameters of TA of **DPyA** and **DPyN** in toluene. All parameters are extracted from the global fitting analysis. Table S2: The effective NLA coefficients of twistacenes **DPyA** and **DpyN** at selected wavelengths.

## Sample Availability

Samples of the **DPyA** and **DPyN** are available from the authors.
