# Supplementary material for: Enhanced Ultrafast Broadband Reverse Saturable Absorption in Twistacenes with Enlarged π-Conjugated Central Bridge
Source: Molecules. 2022 Dec 19;27(24):9059. doi: 10.3390/molecules27249059 (PMC9782705; doi:10.3390/molecules27249059)
Supplement: Supplementary file 1 [file molecules-27-09059-s001.zip › molecules-2096006-supplementary.pdf]

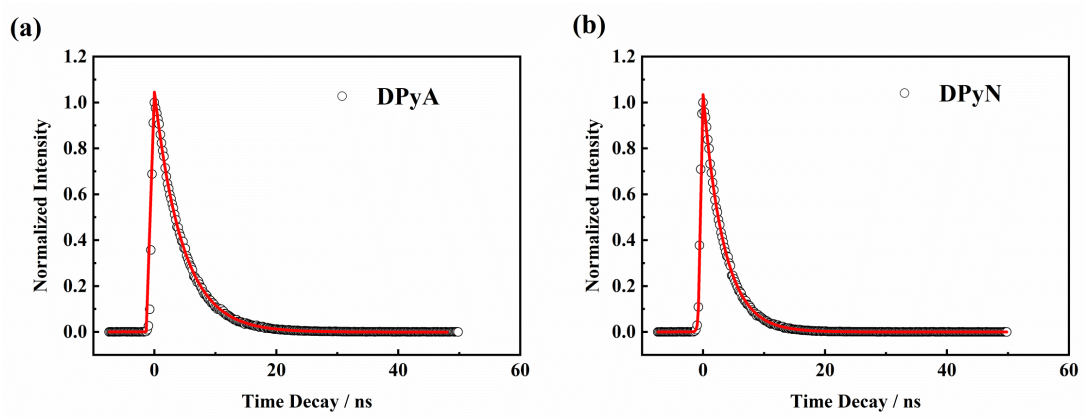

Figure S1. Fluorescence decay data of **DPyA** (a), DPyN (b) by TCSPC. The solid lines were fitted lifetimes of 4.6 ns for **DPyA** and 3.4 ns for **DPyN**.

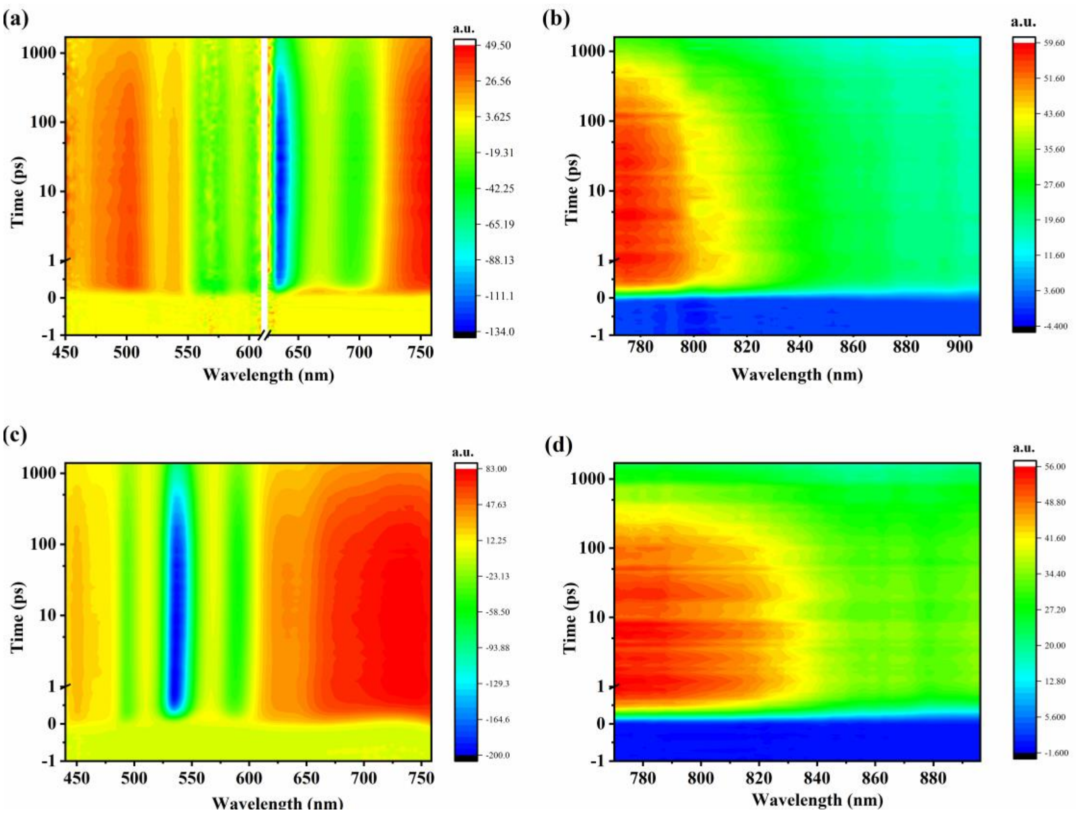

Figure S2. The TA spectra of **DPyA** at the visible window (a) and near the infrared window (b) excited at 400 nm. The TA spectra of **DPyN** at the visible window (c) and near the infrared window (d) excited at 400 nm.

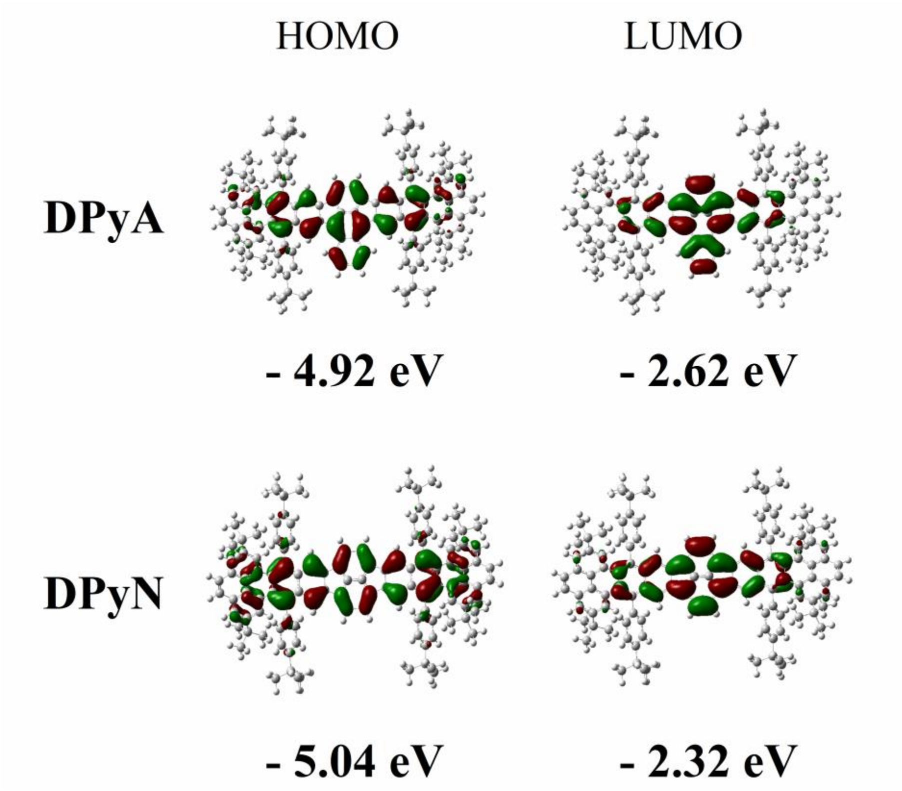

Figure S3. The frontier molecular orbital distributions of **DPyA** and **DPyN** extracted from DFT calculation.

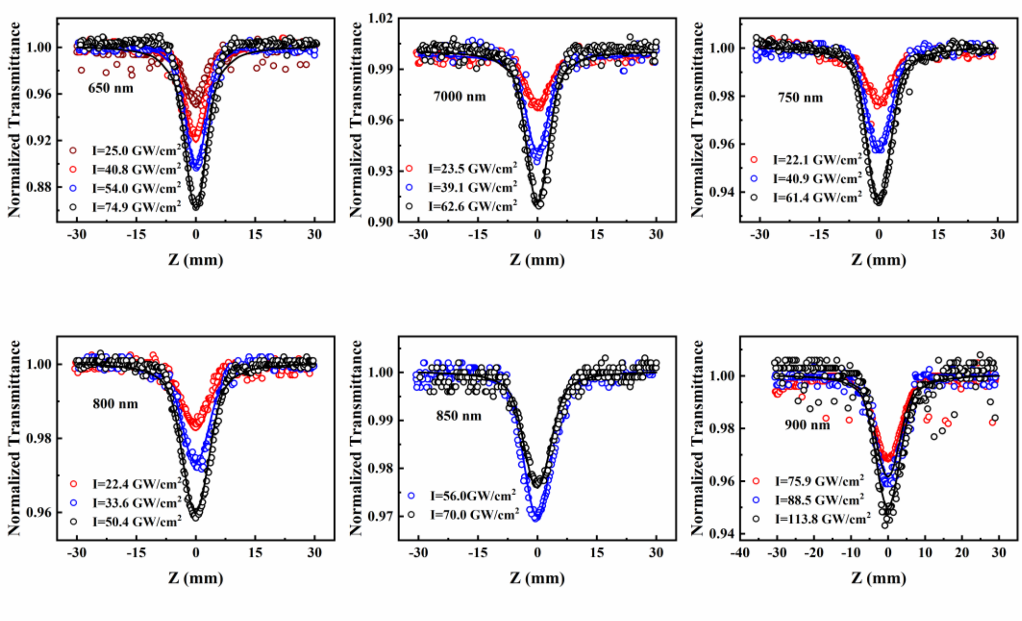

Figure S4. Open-aperture Z-scan results of **DPyA** at different excitation wavelengths (650 nm, 700 nm, 750 nm, 800 nm, 850 nm and 900 nm).

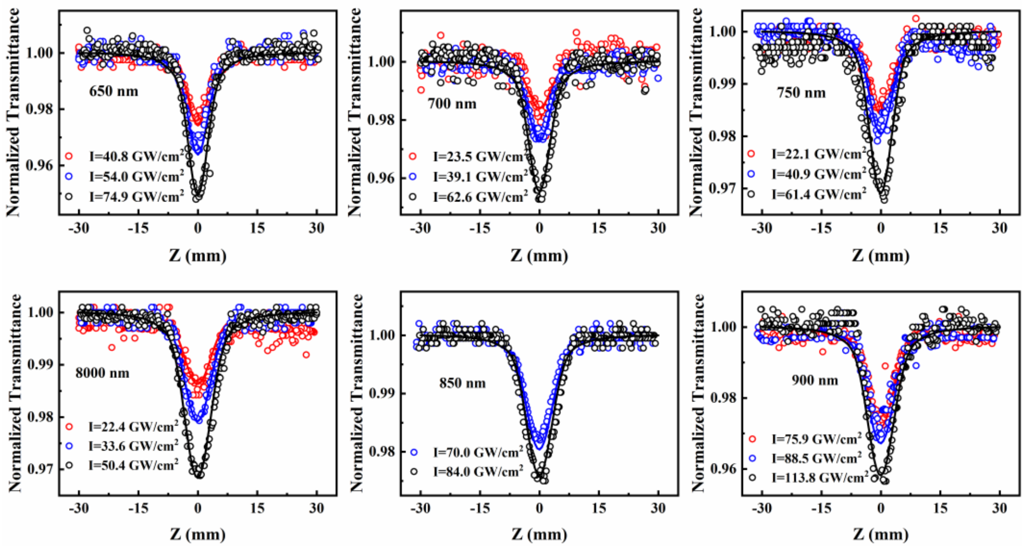

Figure S5. Open-aperture Z-scan results of **DPyN** at different excitation wavelengths (650 nm, 700 nm, 750 nm, 800 nm, 850 nm and 900 nm).

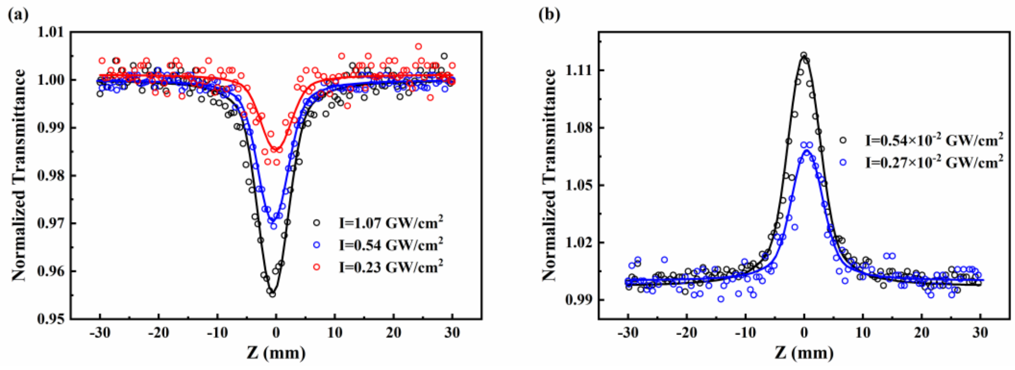

Figure S6. Open-aperture Z-scan experiment at 532 nm under 22 ps laser width for **DPyA** (a) and **DPyN** (b).

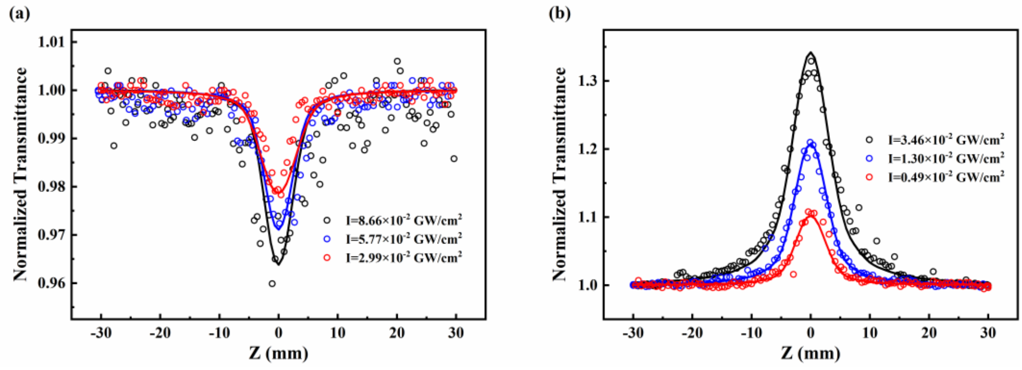

Figure S7. Open-aperture Z-scan experiment at 532 nm under 4 ns laser width for **DPyA** (a) and **DPyN** (b).

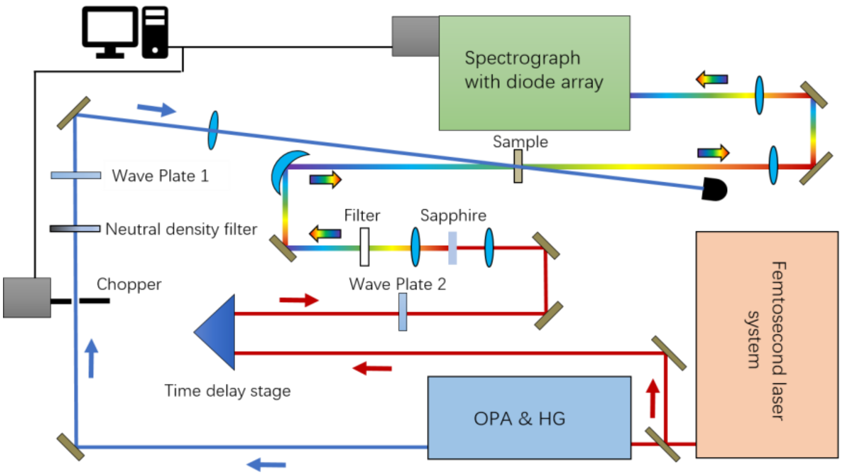

Figure S8. Schematic illustration of transient absorption system.

The output light beam of OPA, adjusted for appropriate attenuation and polarization direction, acts as the pump beam for this spectrum. After the probe beam passes through the time delay device, it focuses on the sapphire and generates supercontinum white-light. After collimating and eliminating the influence of fundamental frequency light, the sample is focused with probe beam. The position of the optical delay device is controlled to change the relative difference of optical path between the probe beam and the pump beam, and the excited-states absorption is measured at a specific delay time. The above operation can be repeated repeatedly to see the absorption spectrum changes in the whole excited state process.

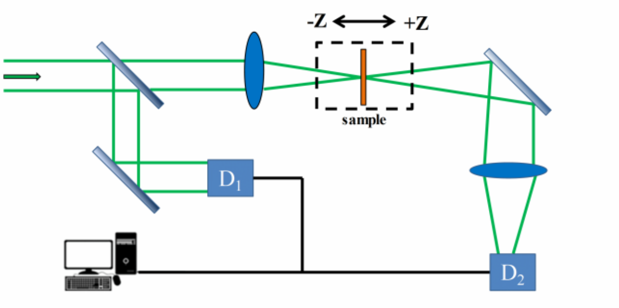

Figure S9. Schematic illustration of z-scan system.

The sample is placed on a linear displacement platform with computer control. The platform drives the sample along the beam propagation direction (Z axis) near the light beam focus. The two probes record the changes of the laser pulse energy passing through the sample and the fluctuation of the laser pulse energy as the sample moved along the z axis, respectively. The incident laser energy is controlled by neutral density attenuation plates. Both the displacement platform and the energy probe are controlled by computer, and the data is recorded while the sample is moving.

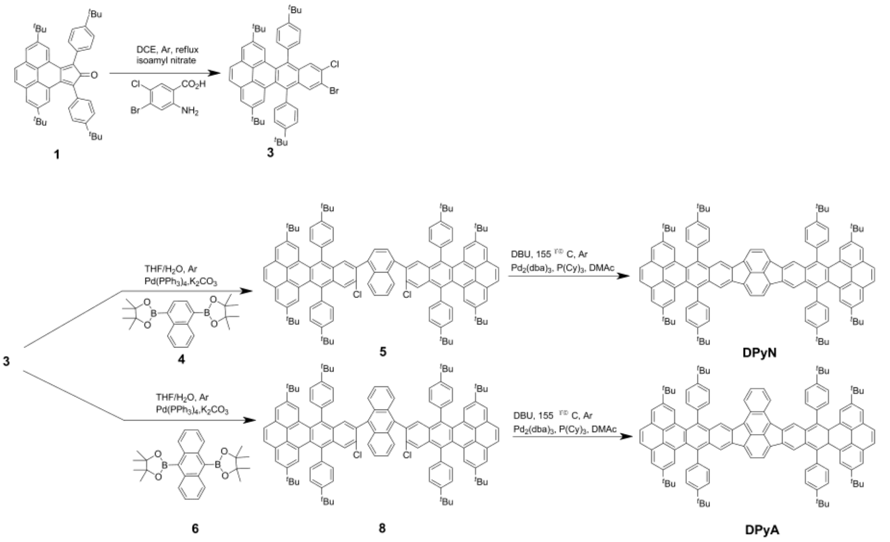

Scheme S1. Synthetic Route to Molecules **DPyA** and **DPyN**.

Table S1. Fitting parameters of TA of **DPyA** and DPyN in toluene; the parameters are extracted from the global fitting analysis.

Sample	τ_1(ps)	τ_2(ps)	τ_3(ns)
**DPyA**	<0.25	4.01	>3.00
**DPyN**	<0.25	2.16	>2.17

Table S2. The effective two-photon absorption coefficients of compounds **DPyA** and **DPyN** at selected wavelengths.

Wavelength	I	**DPyA**	**DPyN**
(nm)	(GW/cm^2)	β_{eff} (10^{-2} cm/ GW)	β_{eff} (10^{-2} cm/ GW)
650	40.8	4.8	1.3
750	40.9	2.6	1.2
800	33.6	1.8	1.3
